# Shared and differentiated motor skill impairments in children with dyslexia and/or attention deficit disorder: From simple to complex sequential coordination

**DOI:** 10.1371/journal.pone.0177490

**Published:** 2017-05-19

**Authors:** Marie-Ève Marchand-Krynski, Olivier Morin-Moncet, Anne-Marie Bélanger, Miriam H. Beauchamp, Gabriel Leonard

**Affiliations:** 1 Research center in neuropsychology and cognition (CERNEC), Université de Montréal, Montréal, Québec, Canada; 2 Ste-Justine Hospital Research Center, Montréal, Québec, Canada; 3 Department of Psychology, Université de Montréal, Québec, Canada; 4 Montreal Neurological Institute and Hospital, Montreal, Quebec, Canada; Universita degli Studi di Roma La Sapienza, ITALY

## Abstract

Dyslexia and Attention deficit disorder (AD) are prevalent neurodevelopmental conditions in children and adolescents. They have high comorbidity rates and have both been associated with motor difficulties. Little is known, however, about what is shared or differentiated in dyslexia and AD in terms of motor abilities. Even when motor skill problems are identified, few studies have used the same measurement tools, resulting in inconstant findings. The present study assessed increasingly complex gross motor skills in children and adolescents with dyslexia, AD, and with both Dyslexia and AD. Our results suggest normal performance on simple motor-speed tests, whereas all three groups share a common impairment on unimanual and bimanual sequential motor tasks. Children in these groups generally improve with practice to the same level as normal subjects, though they make more errors. In addition, children with AD are the most impaired on complex bimanual out-of-phase movements and with manual dexterity. These latter findings are examined in light of the Multiple Deficit Model.

## Introduction

Dyslexia and Attention Deficit, with or without Hyperactivity Disorder (ADHD), are common neurodevelopmental conditions in childhood and adolescence. Prevalence in school age children ranges from 5% to 15% and 5.9% to 7.1% in the United States, respectively [1, 2]. In addition, 60% to 80% of children with ADHD or Dyslexia have a comorbid diagnosis, with 25% to 40% of children meeting the criteria for both conditions [3, 4].

### Dyslexia and motor functioning

Dyslexia is characterized by a failure to attain efficient reading skills despite adequate intellectual abilities and sufficient instruction [5]. Its phenotypic expression varies according to the severity of the reading impairment, the type of errors made (phonological and/or visual memory), and the presence of other impairments in writing and reading comprehension [6, 7]. Several theories have been proffered to explain the mechanisms underlying dyslexia. Primary amongst these is the “Phonological Deficit Hypothesis”, which posits inefficient phonological representations linked to deficient phoneme-grapheme associations [5]. Other cognitive problems often coexist, for instance with working memory [8, 9], prompting recent studies to articulate new theories that attempts to account for the plethora of observed symptoms by invoking impairments in the auditory, visual, and motor domains [10–12]. In keeping with this conceptualization, dyslexia can be viewed as a multifactorial entity where associated cognitive problems are thought not to be limited to language brain areas, nor solely to a dysfunctional phonological system, but rather to multifocal cortical systems [13, 14]. Neuroanatomical and diffusion tensor imaging (DTI) findings support this perspective by identifying widespread structural and functional discordances in dyslexic brains compared to healthy control subjects, including in the occipitotemporal cortex, in the cerebellum and in the bilateral pars triangularis [15–18]. The Cerebellar Deficit Theory of Dyslexia (CDTD) was derived from these observations in an attempt to unify the multiple functional impairments observed in Dyslexia [19]. This theory suggests that impairments can be attributed to a failure of skill automatization leading to information processing and motor skill impairments that can be linked to cerebellar dysfunction. Thus, the CDTD predicts inefficient implicit motor sequential learning and procedural skills in subjects with dyslexia. These findings are further substantiated by Serial Reaction Time Task (SRTT) studies that show procedural learning deficits in dyslexic populations [20–22]. Although support for the CDTD has been inconsistent, the theory underscores the importance of cerebellar-cortical interactions in explaining functional symptoms of dyslexia by taking into account motor impairments [23, 24].

Dyslexic children were shown to be motorically less adept than their typically developing peers, however results across studies have been inconsistent, in part due to methodological differences. Deficits that are more consistently reported in children with dyslexia are with gross motor skills, more specifically balance and postural tasks [25–31]. These studies often support the CDTD hypothesis because of the cerebellum’s known implication in balance skills [32]. Studies also suggest fine motor skill impairments, for instance with peg moving or bead threading [25, 26, 30], though results are more variable in the literature. The inconsistencies found are often associated with the presence of comorbidities in the dyslexia groups, including attention deficit disorder, developmental coordination disorder (DCD) or language disorders [25, 27, 29, 33]. For instance, a study found that motor speed (finger tapping) and peg moving impairments are associated to language impairments rather than dyslexia per se [34]. Further, dyslexic populations’ performances have been shown to be influenced by task complexity. For example, dyslexics were impaired on tasks combining speed and accuracy performances but not when speed was measured alone, or on a task demanding complex out-of-phase coordination of fingers in comparison to less complex synchronized movements [35–38]. However, to our knowledge, a limited number of findings address the impact of task complexity on motor performances among dyslexic populations. Finally, studies on procedural learning of sequences with a Serial Reaction Time (SRT) paradigm have supported the presence of automatization and procedural memory impairments in dyslexic populations [39]. Interestingly, some of the impairments reported in children with dyslexia included sequential movements, for example finger to thumb, though few studies outside of the SRT paradigm have specifically measured sequencing skills. Employing assessment techniques that sample fine and gross motor abilities, ranging from simple motor speed, to sequential motor skills and to complex coordination would contribute to resolving these discrepancies.

### ADHD and motor functioning

Attention Deficit/Hyperactivity Disorder (ADHD) is characterized by developmentally inappropriate inattentiveness, impulsivity and hyperactivity with consequent impairment in academic achievement and reduced success in every-day life [40]. The Diagnostic and Statistical Manual of Mental Disorders, Fifth Edition (2013) lists the following ADHD specifications: a) primarily inattentive; b) primarily hyperactive/impulsive; and c) combined type, however, controversy remains on the cataloguing of differences in the phenotype definition of ADHD [41–44]. For the purpose of the present study, the term AD will be used to avoid characterizing differences in phenotype definition across studies, the main purpose being to evaluate motor skills in relation to a general Attention deficit disorder with or without a hyperactivity diagnosis.

Children with AD show deficits on different neuropsychological measures, including vigilance, working memory, response inhibition, set shifting, planning and motor control [45–47]. Barkley’s model of Attention Deficit Disorder suggests that AD is linked to inefficiencies in internalizing sensory input (internally represented information) that then lead to deficient motor control as well as a lack of efficacy in different cognitive capacities that include motor inhibition and the execution of complex motor sequences requiring flexibility (i.e. fluency such as writing and drawing) [48, 49]. Identification of a core deficit remains elusive, which has encouraged studies on neuroanatomical differences in AD populations. Various studies have focused on frontal lobe involvement, which might account for the surfeit of cognitive difficulties mentioned above [50]. Frontal lobe dysfunction in AD is associated with smaller brain volumes in this region and altered activity in the anterior cingulate, dorsolateral, and ventrolateral prefrontal cortex, as well as in associated striatal, parietal and cerebellar regions; these later findings may suggest abnormal functioning of fronto-striatal and fronto-parietal neural circuitry [51–53]. Interestingly, the frontal lobe has been associated to «higher order» motor skills, namely bimanual coordination [54, 55].

The presence of motor impairment in AD populations is widely reported. Several studies have readily distinguished fine and gross motor skills and suggested impairments in both domains [56–60]. Some of these authors have also suggested that the complexity of the task influences the degree of impairment, for instance by showing that more complex upper limb tasks accentuate differences between children with AD and their typically developing peers [56, 60]. In addition, a study reported that children with AD make significantly less accurate movements than control groups with in-phase and out-of-phase bimanual coordination, further substantiating the link between frontal-lobe dysfunction and bimanual coordination impairments [54, 61]. Procedural learning difficulties and sequential movement deficits in AD populations have also been reported [62–64]. However, debate is still present on the etiology of these impairments, for example whether they emerge from attentional problems, inhibition impairments or the presence of comorbid diagnosis, such as DCD.

### Comorbid AD and dyslexia

AD and dyslexia share high comorbidity rates and are both characterized by cognitive and motor skill impairments [12, 65, 66]. Some authors suggest that it is the presence of co-morbid disabilities that account for shared symptoms, for instance subjects with dyslexia who have motor skill deficits have comorbid high attention deficit ratings which explains shared symptoms rather than dyslexia *per se* [11, 27]. However, the overlap of impairments between disorders such as dyslexia and AD have led others to postulate a unifying framework for complex disorders. Theories such as the Atypical Brain Development theory (ABD) [67] or the Multiple Deficit Model [65, 68] acknowledge the underlying nonspecificity of the mechanisms responsible for the various and shared symptoms in neurodevelopmental disorders. Rather than hypothesizing that a single neurocognitive deficit could provide a sufficient explanation for the plethora of symptoms, they suggest that neurodevelopmental disorders are heterogenous and that their etiology is multifactorial and originates from the interactive effects of genes and environmental risk factors [12, 65, 69]. Not surprisingly, motor functioning is frequently impaired in heterogeneous neurodevelopmental disorders like dyslexia and AD, conceivably because of its associated elaborate neural networks that are often included in the widely distributed neural correlates of each disorder [70, 71]. However, comparing these disorders on motor performance has received less attention and this is particularly so for those children who have a comorbid diagnosis. It could help shed light on individual motor performances, as wells as the hypothesis of an interactive effect when both disorders are present.

### Objectives and hypothesis

The primary goal of this study is to assess and compare a range of motor skills in children with Dyslexia alone, AD alone, AD/Dyslexia, and their typically developing peers (control groups). More specifically, we assessed fine and gross motor skills ranging from simple speed, through unimanual sequential movements, to complex bimanual coordination. We predict that given the lack of reported simple motor speed deficits, subjects with dyslexia and AD or both, will not be impaired on motor tasks that require a simple speeded repetitive movement. However, given the reported sequencing and gross motor deficits in both populations, we predict that impairment will be apparent on the gross unimanual sequential conditions in the three clinical groups. In addition, given the added complexity of bimanual coordination, our clinical groups will be impaired on these conditions. However, given the findings on bimanual coordination and its link to frontal-based mechanisms, we expect that subjects who have AD and AD/Dyslexia will demonstrate significantly greater impairment than Dyslexia participants. A second goal is to identify differences in sequential motor skill acquisition as determined by examining practice effects from Trial 1 to Trial 2 because of the reported deficits in sequential skill learning in both populations. We hypothesize that our three clinical groups will show less efficient sequential skill acquisition as evidenced by lower improvement scores from Trial 1 to Trial 2 than the control groups. A third goal is to measure fine motor skills. Given the inconsistent results with manual dexterity in dyslexic populations, we predict that the dyslexia group will not be impaired because of the relative simplicity of the chosen task. Contrariwise, impairments will be present in the AD and Combined groups because of the more consistently reported fine motor skill impairments in populations with attention deficit disorders. Finally, we measure accuracy by comparing performance errors made on the different conditions. We predict that the experimental groups will make more errors than the control groups, though it is an open question as to what type of errors will characterize performances.

## Materials and methods

### Participants in the experimental groups

Twenty-seven children with dyslexia (DYS, 16 males; M_age_ = 13.8; SD = 2.4 years), 27 children with Attention Deficit with or without Hyperactivity Disorder (AD; 20 males; M_age_ = 12.7; SD = 2.4 years) and 27 children with combined AD and dyslexia (COMB; 19 males; M_age_ = 12.7; SD = 2.6 years) were recruited in the context of a larger study on learning disabilities at a special education school for French and English speaking children with learning disabilities in Montreal, Quebec. See Table 1 for participants’ demographic and characteristics. Children are admitted to this school if they are two years behind in specific classes (French/English and arithmetic classes) and if they have diagnosed learning impairments (reading, writing, executive functions, etc.) with normal or superior intellectual abilities. All participants and their legal guardian gave written informed consent to participate in this study approved by the Montreal Neurological Institute (McGill University) and compliant with the 1964 Declaration of Helsinki.

**Table 1:** Descriptives.

Groups	Age*	TONI-4*	Handedness*	WIAT-II*		Conners (Parent)*		Other problems
				*Reading*	*Spelling*	*Inattention*	*Hyperactivity*	
**DYS** (n = 27)	13.78	97.85	24.81	83.22	71.7	54.96	50.73	BC *(n = 2); APD (n = 3);*
(16 males)	*2*.*38*	*8*.*05*	*12*.*43 *	*11*.*69*	*7*.*7*	*10*.*21*	*9*.*33*	*LT(n = 1); P (n = 1)*
**C1** (n = 27)	13.78	97.74	24.33	-	-	-	-	BC *(n = 2)*
(16 males)	*2*.*37*	*7*.*16*	*14*.*5*					
**AD** (n = 27)	12.59	97.9	33.15	98.11	80.59	73.82	68.33	BC *(n = 4); APD (n = 4);*
(20 males)	*2*.*21*	*9*.*7*	*22*.*33*	*11*.*28*	*12*.*1*	*11*.*61*	*15*.*21*	*LT (n = 5); P (n = 3)*
**C2** (n = 27)	12.59	100.11	30.44	-	-	-	-	LT *(n = 1);* Hypo. *(n = 1)*
(20 males)	*2*.*21*	*9*.*62 *	*20*					
**COMB** (n = 27)	12.63	99.59	25.78	82.89	74.37	70.37	63.67	LT*(n = 3);* P *(n = 2)*
(19 males)	*2*.*57*	*11*.*24*	*11*.*95 *	*16*.*11*	*10*.*43*	*11*.*89*	*14*.*77*	
**C3** (n = 27)	12.63	100.9	23.37	-	-	-	-	-
(19 males)	*2*.*57*	*10*.*94 *	*9*.*73 *					

BC, Birth complications; APD, Auditory Processing Disorder; LT, Late talking; P, Premature; Hypo., Hypothyroidism.

*Means and standard deviations (SD).

Children were included in the AD group if they had a previous clinical diagnosis of AD as reported in the medical questionnaire completed by parents. They were excluded if they had a history of dyslexia. Children were included in the dyslexia group if they had a previous clinical diagnosis of dyslexia, in the absence of AD (See *Clinical measures* section below). Children were included in the combined AD/dyslexia group if they had previous diagnoses of AD and dyslexia.

Exclusion criteria for all groups were: 1) Documented medical history of learning disabilities other than AD or Dyslexia (dysphasia, dyspraxia and dyscalculia), traumatic brain injury, neurological or psychiatric conditions, or an IQ rating below 80.

### Control participants

Each clinical group was compared to 27 typically developing children (DYS/C1, AD/C2, COMB/C3) matched for age (within a year), gender, handedness, and IQ (M = 100; IQ +/**-** 20 points) (Dyslexia (C1): 16 males; M_age_ = 13,8; SD = 2,4; AD (C2): 20 males; M_age_ = 12,7; SD = 2,4; Combined (C3): 19 males; M_age_ = 12,7; SD = 2,6). 81 Control participants were selected from a bank of 1800 participants aged 6 to 95 years old recruited and tested in the context of a larger study on motor skills in children and adolescents [72]. The exclusion criteria for this group were: 1) presence of learning disabilities 2) premature birth (less than 37 weeks’ gestation) 3) traumatic brain injury or 4) neurological/psychiatric conditions.

A limited number of participants was included in our experimental groups for matching purposes; the groups also include participants with other birth complications or co-morbidities (Table 1). We believe however that our sample remains representative of the population considering the high prevalence of other complications or disabilities in children with dyslexia or AD.

### Clinical measures

The following tasks were included in the protocol for descriptive purposes and to verify inclusion and exclusion criteria in the clinical groups.

TONI 4 (Test of non-verbal intelligence, Fourth Edition): This test was designed to assess non-verbal general intelligence while avoiding the confounding effects of a person’s linguistic or motor skills on global performance in individuals aged 6 to 90 years old. The TONI 4 taps into the capacity to reason abstractly while minimizing cognitive abilities such as reading, writing speaking or listening. The examinee must focus on differences and similarities among abstract/figural content, identifying the rule or rules to find the correct response [73].

Medical- Educational-Social Questionnaire: A custom medical questionnaire from the Montreal Neurological Institute was used to collect information on participants’ medical and developmental history including information on previous diagnoses of AD, language and learning disabilities, medical and psychiatric disorders, and history of traumatic brain injury. Other information included socio-economic status, parental education, treatment or remediation received and medication.

Wechsler Individual Achievement Test, Second Editio*n* [74]: This standardized assessment battery measures academically linked skills as well as functions typically affected by learning disabilities. *Pseudo-Word Decoding* and *Spelling* subtests were used to support the diagnosis of dyslexia. In the *Spelling* subtest, the examinee must write dictated letters, letter blends and words. The *Pseudo-Word Decoding* subtest evaluates the ability to apply phonetic decoding skills. The examinee must read aloud a list of nonsense words designed to imitate the phonetic structure of words in the English or French language. Reading and writing disability were defined as a standard score below 80 on these subtests.

Conners 3- Parent forms [75]: This behavioral questionnaire measures the presence of attention deficit/hyperactivity disorder and associated problems and disorders. It features multiple content scales that assess ADHD related problems such as inattention, hyperactivity, executive functioning, learning, aggression, and peer/family relations. These are summed into an ADHD and ADD Index score as well as a Global Index score. The ADHD and ADD Index scores (Mean = 50, SD = 10) were used to support the diagnosis of AD, as defined by a standard score over 60 (borderline range or higher).

### Measures of motor performance

Handedness questionnaire: We used a modified version of the Edinburgh Handedness Inventory [76] that includes eighteen questions about stated hand preference for eighteen actions (e.g. throw a ball) to which individuals can answer «right hand always», «right hand most of the time», « both hands equally often», «left hand most of the time» or «left hand always». The total possible score is 90. An individual with a score of 18 to 29 is considered right-handed, 29 to 54 ambidextrous, and 55 to 90 left-handed. For the purpose of this study, we used the terms Dominant Hand (DH) and Non-Dominant Hand (NDH).

Grooved Pegboard (GPB) [77]: This well validated dexterity test was used to measure fine motor skills. There are 25 key shaped holes in which the subject must insert one peg at a time. All the pegs have a round side and a square side and must be rotated to match each hole on the pegboard before they can be inserted. There are five rows of five holes each that need to be filled twice (*In* condition) with each hand, as quickly as possible, starting with the dominant hand (i.e., *dominant*—*non-dominant*—*non-dominant*—*dominant*). Pegs are inserted from *left to right with the right hand*, *and vice versa for the left*. In addition, the subject is timed for taking the pegs out (*Out* condition) after each trial from the bottom (*Right hand*: *right to left and left hand*: *left to right*). A time score is computed for each hand on each trial.

**Fig 1:**
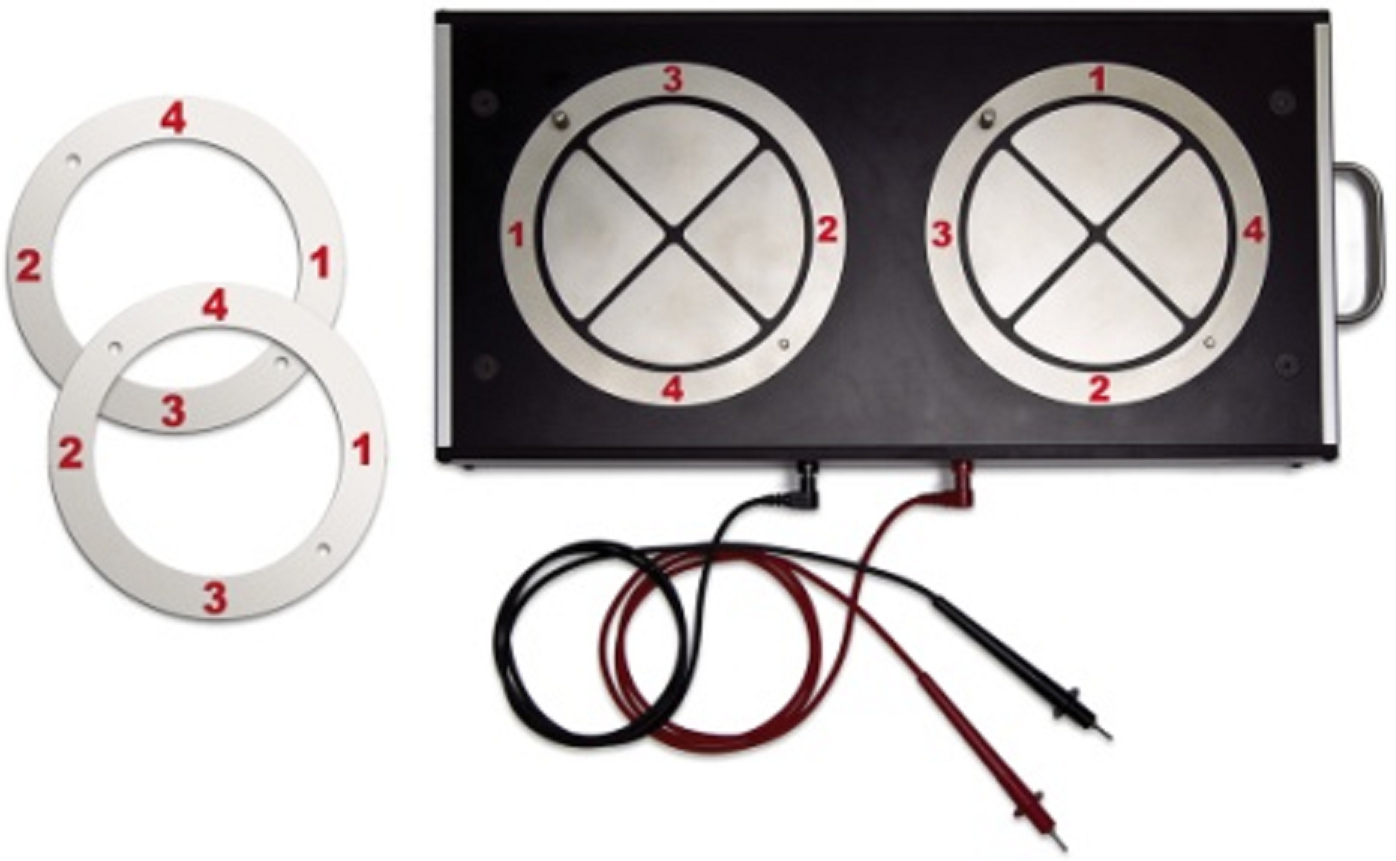
The Leonard Tapping Task (LTT).

Leonard Tapping Task (LTT; Fig 1): This task was designed to rapidly assess simple (i.e. unimanual rapid tapping) and complex motor (i.e. bimanual out of phase movements) coordination and motor sequencing. It is being validated on 1800 participants aged 6 to 95 years [72]. The task is a modified computerized version of the Thurstone apparatus [78], designed to measure both unimanual and bimanual coordination. The Thurstone apparatus and its adaptation (LTT) have been used extensively in a clinical setting (Montreal Neurological Institute), as well as in studies on motor coordination [54, 79]. The LTT is composed of two symmetrical round metal plates with 4 equally sized quadrants numbered from 1 to 4 on which the subject must tap with a stylus following a sequential order with either one hand at a time or both hands together. Four conditions are administered and conditions 1, 2 and 3 are repeated twice after the first three trials are completed, without any time interval in between. Condition 4 is performed after both trials of conditions 1 to 3 are achieved.

1. Unimanual Sequential Tapping (UniSeq): The subject holds a stylus in his right hand or left hand and taps on the metal plates sequentially in a numerical order (1, 2, 3, 4) and continues the sequence for 30 seconds as fast as possible. The participants begin the first trial using their dominant hand, and the second using the non-dominant hand.

2. Bimanual In Phase (or balanced) Tapping (BiBal): Rings are positioned around each plate in order to change the numbers on the quadrants (hence they are in corresponding positions for both hands). The subject holds a stylus in each hand and must tap simultaneously both 1s, 2s, 3s an 4s, and continue sequentially as fast as possible for 30 seconds.

3. Bimanual Out of Phase (or unbalanced) Tapping (BiUnbal): The rings previously set for the BiBal condition are removed (hence the numbers of each plate are not in corresponding positions). The subject holds a stylus in each hand and must simultaneously tap both 1s, 2s, 3s and 4s, and continue sequentially as fast as possible for 30 seconds.

4. Rapid Repetitive Tapping (RT): The subject holds a stylus in the right hand or left hand and taps on a metal plate (quadrant 4 with the left hand and quadrant 2 with the right hand) as fast as possible for 15 seconds with each hand, starting with the dominant hand. The measure consists in the total number of taps by each hand.

Five error types were documented: *Sequential*, *Omission*, *Perseverative*, *Unimanual* and *Balance* errors:

1. Unimanual Sequential Tapping: *Omission*, *sequential* and *perseverative* errors are recorded. An *Omission* is recorded if the subject taps with the stylus on any quadrant on the non-designated side. A *sequential* error is recorded if the subject taps with the stylus on a quadrant on the designated plate that does not follow the numerical order of the sequence (1, 2, 3, 4). A *perseverative* error is recorded if the subject taps with the stylus on the same quadrant a second consecutive time.

2. Bimanual Tapping: For both In Phase and Out of Phase tasks, all error types are possible. An *omission* error occurs when the subject taps on only one of the two metal quadrants or if the subject taps with both hands on the same quadrant. A *perseverative* error is recorded if the subject taps again on the same quadrant with one or both hands. A *sequential* error is recorded if the subject taps with both hands on any other quadrant that does not follow the sequence. If the subject taps on two quadrants that do not correspond and neither one corresponds to the right sequence, a *sequential* error is recorded. A *balance* error is recorded when one hands taps on the correct quadrant (sequentially ordered) but the other hand taps on a non-corresponding quadrant. A *unimanual* error is recorded when one of the hands fails to tap.

3. Rapid Repetitive Tapping: Only *Omission* errors are recorded. They are counted when a subject does not touch the designated metal quadrant, but rather contact is made outside of the rings or with another quadrant.

### Data analyses

Age, gender, IQ and handedness score were compared between the three experimental groups (DYS; AD; COMB) and their matched control group (C1; C2; C3) using a one-way ANOVA for each variable.

**Fig 2:**
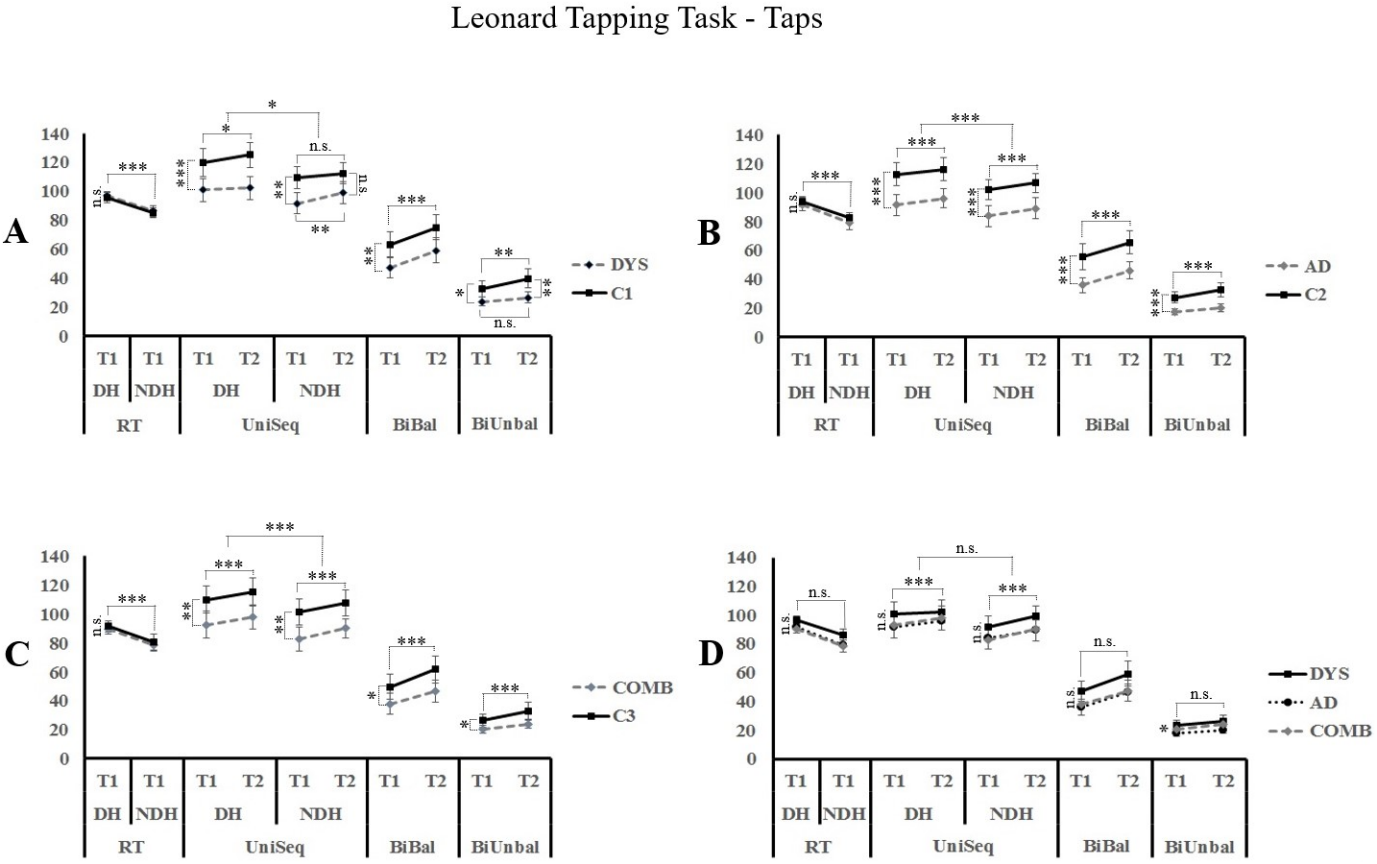
Motor performances on the Leonard Tapping Task (LTT) between experimental groups and control groups. Performances are expressed as the number of correct taps per condition on the LTT between A) dyslexia (DYS) versus controls (C1); B) Attention Deficit Disorder (AD) versus controls (C2); C) Combined Dyslexia and Attention Deficit Disorder (COMB) versus controls (C3); D) Dyslexia (DYS) versus Attention Deficit Disorder (AD) versus Combined dyslexia and Attention Deficit Disorder (COMB). *p < 0.05; **p < 0.01; ***p < 0.001; n.s. = non significant. Only the simple effects are reported when post-hoc analysis are not required. T1 = Trial 1; T2 = Trial 2; DH = Dominant Hand; NDH = Non-Dominant Hand; RT = Rapid Tapping; UniSeq = Unimanual Sequential; BiBal = Bimanual Balanced; BiUnbal = Bimanual Unbalanced.

In order to assess differences in motor performance, the number of correct taps performed on each condition (unimanual or bimanual) of the LTT was measured. Each experimental group was compared to its matched control group on the four LTT conditions (Fig 2). Table 2 indicates the mean performances of the six groups on each condition.

**Table 2:** Leonard Tapping Task mean performances by group and condition (Trials 1 & 2).

Groups	RT*		UniSeq- DH*		UniSeq- NDH*		BiBal*		BiUnbal*	
	DH	NDH	Trial 1	Trial 2	Trial 1	Trial 2	Trial 1	Trial 2	Trial 1	Trial 2
**DYS**	96.73	86.38	100.93	102.44	91.74	99.07	47.26	59.11	23.74	26.59
(n = 27)	*7*.*47*	*10*.*28*	*21*.*43*	*21*	*19*.*4*	*19*.*66*	*18*.*81*	*23*.*27*	*8*.*47*	*9*.*67*
**C1**	95.56	85.3	119.63	125.07	109.33	112.3	63.37	75.04	32.56	39.67
(n = 27)	*8*.*58*	*9*.*43*	*25*.*9*	*22*.*33*	*20*.*45*	*19*.*17*	*22*.*17*	*22*.*57*	*12*.*09*	*17*.*08*
**AD**	91.85	79.37	91.44	96.19	83.85	89.3	35.96	46.19	17.67	20.33
(n = 27)	*12*.*12*	*12*.*82*	*19*.*68*	*17*.*08*	*19*.*29*	*19*.*49*	*14*.*43*	*16*.*13*	*5*.*39*	*6*.*82*
**C2**	93.81	82.67	112.82	116.15	101.93	106.67	55.57	65.59	27.52	32.85
(n = 27)	*8*.*65*	*8*.*76*	*21*.*51*	*20*.*86*	*18*.*45*	*18*.*13*	*24*.*18*	*21*.*23*	*9*.*35*	*13*.*28*
**COMB**	89.96	78.37	92.78	97.7	82.96	90.07	37.89	47	20.56	24.15
(n = 27)	*9*.*11*	*11*.*25*	*25*.*21*	*21*.*97*	*22*.*05*	*17*.*88*	*19*.*38*	*20*.*05*	*6*.*94*	*8*.*82*
**C3**	91.59	80.7	110.04	115.48	101.33	107.59	49.78	61.82	26.3	32.85
(n = 27)	*9*.*78*	*14*.*82*	*24*.*66*	*25*.*81*	*24*.*14*	*23*.*62*	*23*.*63*	*24*.*95*	*11*.*74*	*15*.*82*

* Mean number of taps and standard deviations (SD) of a given Leonard Tapping Task condition are listed.

For the RT condition, a repeated measures 2-way ANOVA was computed with *Hand* (Dominant Hand (DH); Non Dominant Hand (NDH)) as the within-subject factor and *Group* (DYS/ C1; AD/ C2; COMB/ C3) as the between subject factor. For the UniSeq, a repeated measures ANOVA was used with *Hand* (DH; NDH) and *Trial* (T1; T2) as the within-subject factors and *Group* (DYS/ C1; AD/ C2; COMB/ C3) as the between subject factor. For the BiBal and BiUnbal conditions, repeated measures ANOVA with *Trial* (T1; T2) as the within-subject factor and *Group* (DYS/ C1; AD/ C2; COMB/ C3) as the between subject factor were computed.

Performance on each condition (RT; UniSeq; BiBal; BiUnbal) were compared with separate repeated measures ANCOVA with either/or *Hand* (DH; NDH) and *Trial* (T1; T2) as the within-subject factor, *Group* (DYS; AD; COMB) as the between subject factor and *Age* or *Handedness scores* as covariates.

**Fig 3:**
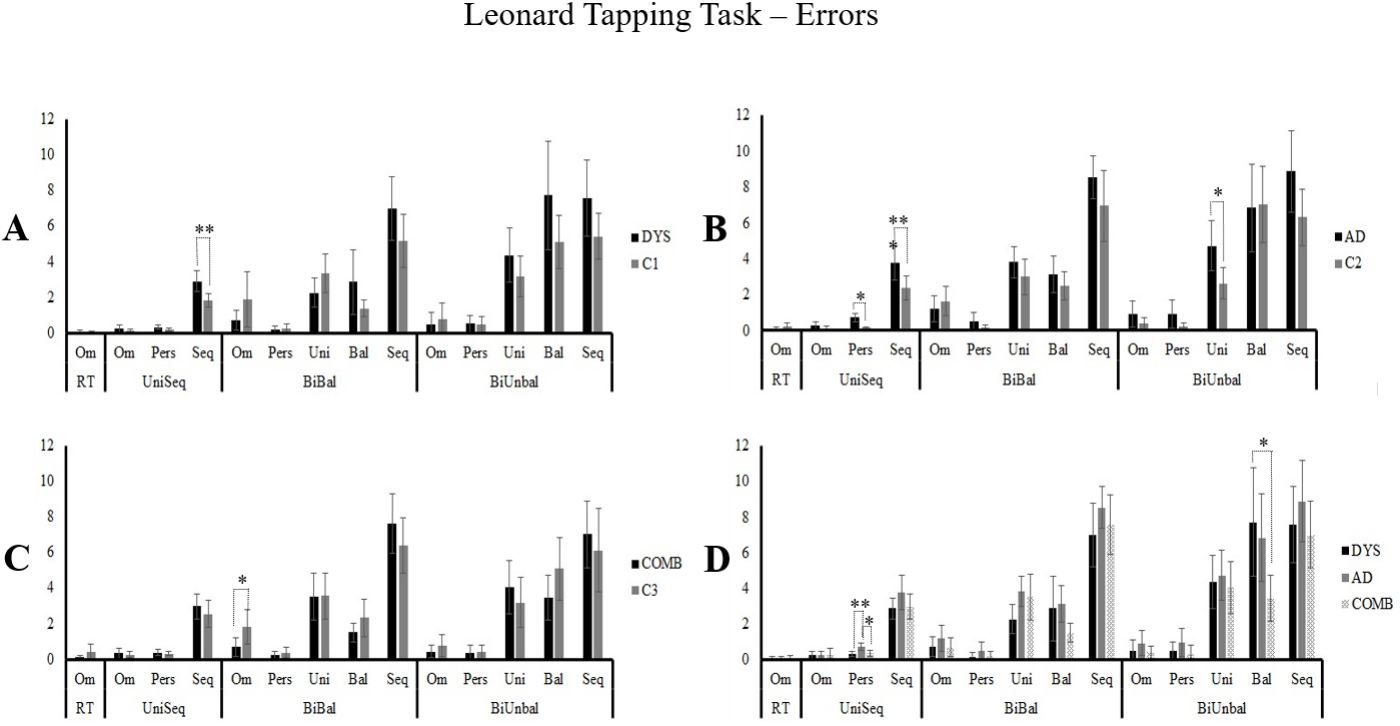
Error ratios on the Leonard Tapping Task (LTT) between experimental groups and control groups. Performances are expressed with the mean ratio of errors per condition, computed with the number of errors of each category (omissions (Om), perseverative (Pers), sequential (Seq), unimanual (Uni), and balanced (Bal)) divided by the number of taps on any given condition of the LTT. Ratios were averaged between the dominant hand (DH) and the non-dominant hand (NDH) for the rapid tapping condition (RT), between the DH and NDH on trial 1 and 2 for the unimanual sequential condition (UniSeq), and between trial 1 and 2 for the bimanual balanced (BiBal) and unbalanced (BiUnbal) conditions. A) dyslexia (DYS) versus controls (C1); B) Attention Deficit Disorder (AD) versus controls (C2); C) Combined Dyslexia and Attention Deficit Disorder (COMB) versus controls (C3); D) Dyslexia (DYS) versus Attention Deficit Disorder (AD) versus Combined dyslexia and Attention Deficit Disorder (COMB). *p < 0.05; **p < 0.01; ***p < 0.001; unreported comparisons are non-significant. Only the simple effects are reported when post-hoc analysis are not required.

Error analyses was conducted using the average number of errors for both hands for the Rapid Tapping (RT). The average number of errors for both hands and both trials was used for the UniSeq condition, whereas the average of both trials for the bimanual conditions was measured. Further, in order to control for motor speed, the number of errors of each type (Om; Pers; Uni; Bal; Seq) was divided by the total number of taps on each condition (RT; UniSeq; BiBal; BiUnbal). As a result, the mean ratio of errors (Fig 3) was compared for each condition between the experimental groups and their respective controls (DYS/ C1; AD/ C2; COMB/ C3) using independent two-tailed T-Tests on all error types across the four conditions. For the comparisons between the experimental groups, the mean ratio of errors of each type (Omission, perseverative, sequential, unimanual and balanced) were compared on each condition (RT, UniSeq, BiBal, BiUnbal) using one-way ANCOVA with *Group* (DYS, AD, COMB) as the between-subject factor and *Age* or *Handedness scores* as covariates. Only the significant effects are reported at a p level of 0.05.

Finally, the performances on the GPB (Table 3) were computed as the time taken to complete 5 rows for the *In* and *Out* conditions separately using repeated measures ANOVA with *Hand* (DH; NDH) and *Trial* (T1; T2) as the within-subject factors and *Group* (DYS/ C1; AD/ C2; COMB/ C3) as the between-subject factor. Furthermore, two separate repeated measures ANCOVA were computed with *Hand* (DH; NDH) and *Trial* (T1; T2) as the within-subject factors and *Group* (DYS; AD; COMB) as the between-subject factor in order to compare performances on both conditions while controlling for *Age* or *Handedness scores*.

**Table 3:** Performances by group and Grooved Pegboard (GPB).

Groups	GPB - 1^st^ Trial*				GPB - 2^nd^ Trial*			
	DH		NDH		DH		NDH	
	IN	OUT	IN	OUT	IN	OUT	IN	OUT
**DYS**	60.92	20.39	65.69	20.73	56.77	18.31	62.62	19.23
(n = 27)	*8*.*65 *	*2*.*15*	*10*.*87*	*2*.*67 *	*7*.*9*	*1*.*88*	*9*.*72*	*2*.*28*
**C1**	58.7	20.26	62.74	20.44	54.07	18	58	19.11
(n = 27)	*12*.*59*	*2*.*77 *	*11*.*24*	*3*.*27 *	*9*.*88 *	*2*.*37 *	*8*.*27*	*3*.*11*
**AD**	71.78	22.26	79.15	22.56	62.67	20.73	72	20.3
(n = 27)	*13*.*78 *	*3*.*58*	*18*.*19*	*3*.*24*	*13*.*79 *	*2*.*67 *	*15*.*07 *	*3*.*04 *
**C2**	63.22	20.63	66.70	21.19	55.96	18.22	61.89	19.44
(n = 27)	*9*.*01 *	*2*.*37 *	*10*.*43*	*2*.*25 *	*7*.*26 *	*1*.*85 *	*8*.*89*	*1*.*85*
**COMB**	64.78	21.41	71	20.3	57.78	18	66.44	19
(n = 27)	*9*.*91*	*3*.*91*	*14*.*72*	*3*.*5*	*7*.*58 *	*2*.*70 *	*13*.*66*	*3*.*46 *
**C3**	60.48	21	64.96	22.22	54.7	20.73	62.41	20.96
(n = 27)	*10*.*92*	*3*.*15*	*11*.*39*	*4*.*26 *	*9*.*97*	*2*.*67 *	*11*.*94*	*3*.*66 *

*Mean completion time and standard deviations (SD) for each hand on the IN and Out conditions of the Grooved Pegboard.

Post-Hoc analyses were conducted accordingly using repeated measures ANOVA for triple interactions, one-way ANOVA and independent T-Tests for group comparisons on single dependent variables and paired T-Tests to measure differences in performances within each group. Bonferroni corrections were used with multiple comparisons. Statistical significance was set at p < 0.05.

## Results

### Group analysis

Table 1 contains the demographic details for the experimental groups and their matched control groups derived from four One-Way ANOVA. Results showed that the groups did not differ significantly on age (F_(5,156)_ = 1.717; p = 0.134), gender (F_(5,156)_ = 0.488; p = 0.765), IQ (F_(5,156)_ = 0.538; p = 0.747) and handedness scores (F_(5,156)_ = 1.645, p = 0.151). More specifically, Post-Hoc Tukey HSD tests indicated that DYS children and their matched control group (C1) did not differ in terms of age (p = 1.00), gender (p = 1.00), IQ (p = 1.00) and handedness score (p = 1.00) and that AD children and COMB children also did not differ from their respective control groups (C2; C3) on all of the variables mentioned above (0.963 ≤ p ≤ 1.000). Parent ratings of Inattention and hyperactivity on the Conners short and long questionnaire for the three experimental groups were analyzed. A simple one-way ANOVA indicated that the groups differed on the Inattention (F_(2,77)_ = 20.890; p < 0.001) and Hyperactivity/Impulsivity scales (F_(2,77)_ = 12.182; p < 0.001). Post-Hoc Tuckey HSD test indicated that the AD and COMB groups did not differ on the Inattention (p = 0.503) and hyperactivity (p = 0.412) scales and were significantly higher than the DYS group on both scales (p < 0.001; p = 0.002). Hence, Parent ratings of AD were in the expected direction on the Inattention and Hyperactivity Scales. Scores on the WIAT-II reading and writing subtests were in the expected direction as well. A simple one way ANOVA indicated that the groups differed on the Spelling test (F_(2,78)_ = 5.360; p = 0.007) and Reading test (F_(2,78)_ = 11.699; p < 0.001). Post-Hoc Tuckey HSD test indicated that the DYS children scored significantly lower on the Spelling (p = 0.006) and Reading (p < 0.001) subtests than the AD children, while the COMB group scored significantly lower on the Reading (p < 0.001) subtest and was marginally significantly lower than the AD group on the Spelling subtest (p = 0.074). DYS and COMBO children did not differ significantly on either subtest (p ≥ 0.606).

As shown in Table 1, the three experimental groups include participants with birth complications, such as emergency c-sections or premature birth, as well as other disabilities such as auditory processing disorders. Other health problems were controlled for as much as possible; however, the reality of “other complications” is an expected finding in these three types of clinical populations.

### Dyslexia group (DYS) vs. control group (C1)

#### Table 2, Figs 2A and 3A

**Fig 4:**
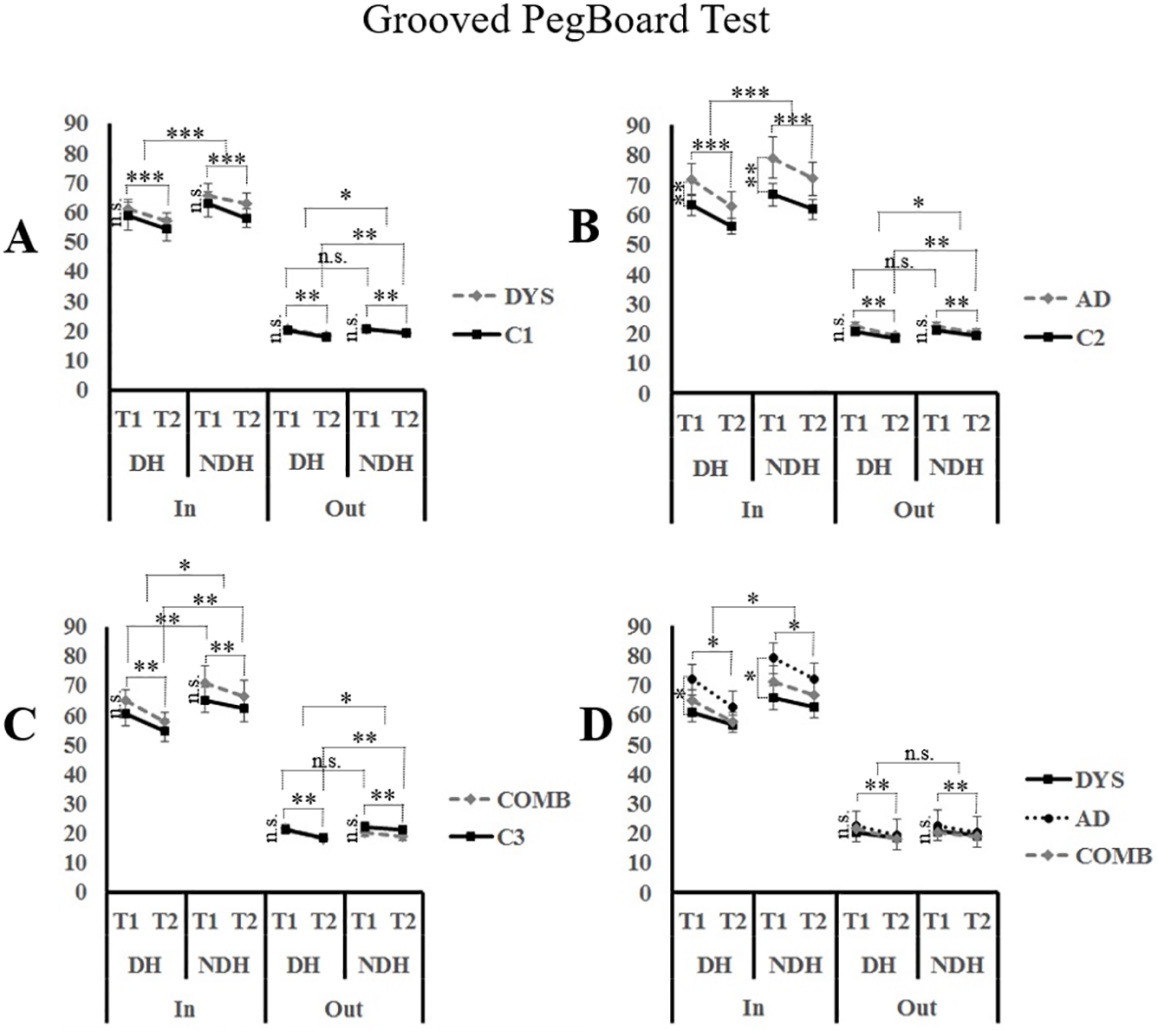
Motor performances on the Grooved Pegboard (GPB) between experimental groups and control groups. Performances are expressed with the completion times on the GPB between A) dyslexia (DYS) versus controls (C1); B) Attention Deficit Disorder (AD) versus controls (C2); C) Combined Dyslexia and Attention Deficit Disorder (COMB) versus controls (C3); D) Dyslexia (DYS) versus Attention Deficit Disorder (AD) versus Combined dyslexia and Attention Deficit Disorder (COMB)*p < 0.05; **p < 0.01; ***p < 0.001; n.s. = non-significant. Only the simple effects are reported when post-hoc analysis are not required.

The number of taps with each hand was compared on the RT condition. Results showed no difference between groups (F_(1,52)_ = 0.272; p = 0.604). Not surprisingly, a significantly higher number of taps (see Figs 1 and 4) was performed with the dominant hand compared to the non-dominant hand (F_(1,52)_ = 82,176; p < 0.001). On the UniSeq condition, results showed a significant *Hand* x *Trial* x *Group* interaction (F_(1,52)_ = 5.286; p = 0.026). Post-hoc analysis included two repeated measures ANOVA with *Trial a*s the within subject factor and *Group* as the between subject factor for the DH and the NDH. Results revealed a main effect of Trial (F_(1,52)_ = 4.867; p = 0.032) and Group (F_(1,52)_ = 11.917; p = 0.001) for the DH, and a significant *Trial x Group* interaction (F_(1,52)_ = 4.231; p = 0.045) for the NDH. Thus, for the DH, both DYS and C1 showed an increase in the number of taps from trial 1 to trial 2, with the DYS group performing more poorly overall. For the NDH condition, post-hoc analyses using paired t-tests with Bonferroni corrections showed a higher increase in performance observed in the DYS group from T1 to T2 (T = -4.08; p < 0.01) with their NDH compared to the C1 group (T = -2.615; p = 0.06), with the DYS group generally completing a lower number of taps on trial 1 (T = -3.243; p = 0.008), but not on trial 2 (T = -2.502; p = 0.064). Comparison of the number of taps on the BiBal condition revealed that both groups increased number of taps on trial 2 compared to trial 1 (F_(1,52)_ = 57.326; p < 0.001) and that the DYS participants make overall significantly less taps than the C1 group (F_(1,52)_ = 7.849; p = 0.007). On a more complex bimanual condition, the BiUnbal, results showed a significant *Trial* x *Group* interaction (F_(1,52)_ = 5.716; p = 0.02). Post-Hoc analysis with Bonferroni corrections revealed that C1 performed an increased number of taps on trial 2 compared to trial 1 (T = -6.407; p < 0.01), while the DYS group’s performance did not improve significantly (T = -2.046; p = 0.204). In addition, the C1 group made overall significantly more taps than de DYS group (T_Trial1_ = -2.787; p = 0.028; T_Trial2_ = -3.461; p = 0.004). Mean error ratios analysis showed that the DYS performed more *sequential* errors compared to their controls on the UniSeq condition (T_(52)_ = 2.89; p = 0.006).

#### Table 3 and Fig 4A

Results on the GPB showed that performance on the *In* condition were greater with the DH compared to the NDH (F_(1,52)_ = 21.501; p < 0.001) and increased from T1 to T2 (F_(1,52)_ = 38.811; p < 0.001) with no groups differences (F_(1,52)_ = 1.696; p = 0.199). Results on the *Out* condition revealed a significant *Hand x Time* interaction (F_(1,52)_ = 5.426; p = 0.024). Bonferroni corrected post-hoc analysis showed that while the performances increased with both hands from T1 to T2 (T_DH_ = 8.563; p < 0.01; T_NDH_ = 6.837; p < 0.01), performances with the DH did not differ from the NDH at T1 (T = -0.820; p = 0.416) but did so at T2 (T = 3.258; p = 0.008).

### Attention deficit disorder group (AD) vs. control group (C2)

#### Table 2, Figs 2B and 3B

On the RT condition, results showed a significantly higher number of taps with the dominant hand compared to the non-dominant hand (F_(1,52)_ = 70.802; p < 0.001) without distinction between groups (F_(1,52)_ = 1.044; p = 0.312). On the UniSeq condition, results revealed that both groups performed more taps with their DH than with their NDH (F_(1,52)_ = 37.634; p < 0.001), and on trial 2 compared to trial 1 (F_(1,52)_ = 24.767; p < 0.001). Furthermore, AD participants made overall significantly less taps than their matched C2 group (F_(1,52)_ = 15.292; p < 0.001). On the BiBal condition, results indicated that both groups made more taps on trial 2 than on trial 1 (F_(1,52)_ = 83.962; p < 0.001), and that AD participants made overall significantly less taps than their controls (F_(1,52)_ = 14.277; p < 0.001). On the BiUnbal condition, results revealed that both groups performed more taps on trial 2 than on trial 1 (F_(1,52)_ = 24.850; p < 0.001), and that AD participants made overall significantly less taps than the C2 group (F _(1,52)_ = 22.183; p < 0.001). Mean error ratios analysis showed that the AD performed more *sequential* (T_(52)_ = 2.304; p = 0.025) and *perseverative* (T_(52)_ = 4.775; p < 0.001) errors compared to their controls on the UniSeq condition, and more *unimanual* errors in the BiUnbal condition (T_(52)_ = 2.502; p = 0.016).

#### Table 3 and Fig 4B

On the GPB, the participants showed increased performances in the *In* condition with their DH compared to their NDH (F_(1,52)_ = 25.14; p < 0.001), and in T2 compared to T1 (F_(1,52)_ = 109.066; p < 0.001). However, the AD participants were slower overall compared to their matched controls (F_(1,52)_ = 10.041; p = 0.003). On the *Out* condition, results revealed a significant *Hand x Trial* interaction (F_(1,52)_ = 4.463; p = 0.039) but no *Group* effect (F_(1,52)_ = 3.895; p = 0.054). Post-Hoc analysis using Bonferroni corrections indicated that the performances with both hands increased from T1 to T2 (T_DH_ = 9.816; p < 0.01; T_NDH_ = 8.678; p < 0.01), and did not differ at T1 (T = -1.189; p = 0.96) but did so at T2 (T = 4.665; p < 0.01).

### Combined group (COMB) vs. control group (C3)

#### Table 2, Figs 2C and 3C

Results on the RT condition showed that the COMB group did not differ from their controls (F_(1,52)_ = 0.541; p = 0.465). However, there was a significantly higher number of taps with the DH compared to the NDH (F_(1,52)_ = 51.204; p < 0.001). On the UniSeq condition, results revealed that both groups made more taps with their DH than with their NDH (F_(1,52)_ = 34.585; p < 0.001) and there was a significant improvement from trial 1 to trial 2 (F_(1,52)_ = 33.053; p < 0.001). COMB participants made significantly less taps than their matched C3 group (F_(1,52)_ = 8.659; p = 0.005). On the BiBal condition, the results revealed that both groups improved from T1 to T2 (F_(1,52)_ = 109.181; p < 0.001). The COMB group performed significantly less taps overall compared to C3 (F_(1,52)_ = 5.058; p = 0.029). On the more complex condition (BiUnbal), the results indicated that both groups made more taps on trial 2 than on trial 1 (F_(1,52)_ = 32.490; p < 0.001) and that COMB participants made overall significantly less taps than the C3 group (F_(1,52)_ = 5.976; p = 0.018). Mean error ratios analysis showed that the COMB group performed fewer *omission* errors compared to their controls on the BiBal condition (T_(52)_ = 2.021; p = 0.048).

#### Table 3 and Fig 4C

On the GPB, performance analysis on the *In* condition showed a significant *Hand x Time* interaction (F_(1,52)_ = 5.36; p = 0.025) without distinction between groups (F_(1,52)_ = 2.491; p = 0.121). Bonferroni corrected post-Hoc analysis indicated that the performances with both hands increased from T1 to T2 (T_DH_ = 9.218; p < 0.01; T_NDH_ = 3.416; p = 0.004), and differed at T1 (T = -4.039; p < 0.01) and T2 (T = 6.567; p < 0.01). On the *Out* condition, a significant *Hand x Time* interaction (F_(1,52)_ = 15.953; p < 0.001) was observed. Post-hoc analysis with Bonferroni corrections revealed that the performances with both hands increased from T1 to T2 (T_DH_ = 9.947; p < 0.01; T_NDH_ = 3.995; p < 0.01), and did not differ at T1 (T = -0.126; p = 0.901) but did so at T2 (T = 5.391; p < 0.004). In addition, the results showed a significant *Hand x Group* interaction (F_(1,52)_ = 10.092; p = 0.003). However, post-hoc analysis using Bonferroni corrections revealed similar performances between both groups with their DH (T = -0.045; p = 0.964) and their NDH (T = -2.085; p = 0.084).

### Experimental groups (DYS vs. AD vs. COMB)

#### Table 2, Figs 2D and 3D

On the RT condition, the results reveal no significant effects of *Hand* (F_(1,77)_ = 3.409; p = 0.069) or *Group* (F_(2,77)_ = 2.853; p = 0.064) after controlling for the *Age* variable. On the UniSeq condition, the participants from the three experimental groups improved on their number of taps from trial 1 to trial 2 (F_(1,77)_ = 13.68; p < 0.001) without differences between *groups* (F_(2,77)_ = 0.134; p = 0.875) or *Hand* (F_(1,77)_ = 1.455; p = 0.231) when controlling for the *Age* factor. On the BiBal condition, the results revealed no differences of *Trial* (F_(1,77)_ = 0.008; p = 0.931) or *Group* (F_(2,77)_ = 1.413; p = 0.25) on the number of correct taps when controlling for *Age*. On the BiUnbal condition, results showed no significant improvement on the number of taps from trial 1 to trial 2 (F_(1,77)_ = 1.582; p = 0.212). However, a significant *Group* difference was observed when controlling for *Age* (F_(2,77)_ = 3.444; p = 0.037). Post-hoc Bonferroni comparisons showed that the AD group (M = 19.695 ± 1.171) performed overall less taps compared to the DYS (M = 23.841 ± 1.186; p = 0.048) group and that the COMB group’s performance (M = 22.983 ± 1.17) did not differ from AD (p = 0.148) or DYS (p = 1.000) groups. Mean error ratio analysis showed significant *Group* effects of *perseverative* errors in the UniSeq condition (F_(2,78)_ = 5.486; p = 0.006) and *balanced* errors on the *BiUnbal* condition (F_(2,78)_ = 3.503; p = 0.035). Post-hoc Bonferroni comparisons show that the AD group performed more *perserverative* errors compared to the DYS (p = 0.009) and COMB (p = 0.024) groups in the UniSeq condition, while the COMB performed fewer *balanced* errors than the DYS group in the BiUnbal condition (p = 0.038).

#### Table 3 and Fig 4D

On the GPB, results on the *In* condition revealed significant *Time x Hand* (F_(1,77)_ = 5.366; p = 0.023) and *Time x Group* (F_(2,77)_ = 4.864; p = 0.01) interactions when controlling for the *Handedness score*. Post-Hoc comparisons of the mean performances estimated with fixed covariates in 95% confidence intervals revealed greater increases in performances with the dominant hand across trials (M_t1_ = 65.826 ± 1.23; M_t2_ = 59.071 ± 1.13) compared to the non-dominant hand (M_t1_ = 71.947 ± 1.663; M_t2_ = 67.02 ± 1.44). Further, the AD group showed overall slower performances but improved significantly more from trial 1 to trial 2 (M_t1_ = 75.625 ± 2.304; M_t2_ = 67.46 ± 2.041) compared to the DYS (M_t1_ = 63.212 ± 2.278; M_t2_ = 59.617 ± 2.018) and COMB groups (M_t1_ = 67.823 ± 2.27; M_t2_ = 62.059 ± 2.011). On the *Out* condition, results indicated that the participant’s speed performances improved from trial 1 to trial 2 (M_t1_ = 21.272 ± 0.305; M_t2_ = 19.034 ± 0.266; F_(1,77)_ = 7.815; p = 0.007) without distinction for the *Hand* (F_(1,77)_ = 1.93; p = 0.169), or *Group* factors (F_(2,77)_ = 2.442; p = 0.094) when controlling for the *Age* variable.

## Discussion

Our study sought to characterize and differentiate motor skill impairments in dyslexia and Attention Deficit Disorder (AD), two prevalent neurodevelopmental disorders that share high comorbidity rates. We confirm that there is a common gross unimanual and bimanual sequential coordination impairment in both disorders. However, neither is compromised on motor speed or motor adaptation, with the exception of the dyslexia group that did not improve significantly on the bimanual out-of-phase condition. Our findings of additional impairments in our AD group on the most complex bimanual coordination task and on manual dexterity are consistent with Pennington’s Multiple Deficit Model [65]. Hence, our data support a unifying framework for the understanding of neurodevelopmental disorders.

Our data make clear that documented motor deficits in dyslexic and AD subjects are not attributable to motor slowness in responding. In keeping with this suggestion, we note that neither group was impaired on rapid unimanual repetitive movements nor in the rapidity with which they could remove pegs from the Grooved Pegboard. We therefore suggest that kinematics and sensorimotor integration are not functionally impaired in these neurodevelopmental disorders. Kinematics include velocity, force, and movement frequencies, and constitute basic components of motor outputs that rely on sensorimotor networks, for instance the primary motor cortex and the supplementary motor areas [55, 80–82].

The addition of complexity that places increased demands on the integration of sequential motor skill and gross coordination, differentiates AD, Dyslexic and Comorbid subjects from their normally developing peers. The motor difficulty expressed by these groups is already apparent when the task is novel and requires spatial-sequential control even in the unimanual paradigm. In addition to performing fewer taps than their respective control peers, the dyslexic group and AD group, were differentiated by their greater propensity to commit sequential errors, suggesting that they had difficulty implementing the appropriate sequence when speed and accuracy were needed. This observation is in keeping with the deficits in sequencing skills reported in both disorders, although they have been reported to a lesser extend in children with AD [39, 83]. Given that these difficulties exist in the unimanual sequential conditions it is not surprising that both in-phase and out-of-phase bimanual coordinated movements are also impaired. Bimanual coordination is considered a «higher order » task because of the added complexity of coordinating both hands [55]. Hence, our findings shed light on shared atypical motor development in all three groups when a task requires gross sequential movements. Our findings also provide insight into the presence of common brain development abnormalities associated with this type of motor output. They have relevance for every-day activities that require elaborate planned sequential movements and require optimal cognitive functioning. Indeed, the fundamental motor impairments expressed by the AD, dyslexic and combined children may have its greatest impact when approaching novel motor tasks; learning situations where cognitive functions, such as attention and working memory are important prerequisites to master a complex motor output [84, 85]. In this regard, widely distributed neural networks are necessary for accurate cognitive-motor output. For example, the cerebellum has been associated with procedural motor abilities as well as with working memory and executive functions [86–88]; and the cingulate cortex and parasagittal frontal regions have been associated with complex motor coordination as well as inhibition and attention [54, 55, 85]. Interestingly, many of these cognitive-motor neural networks have been shown to be functionally depressed in both disorders, namely the prefrontal cortex in AD and the cerebellum in dyslexic populations [18, 50]. Future studies investigating the link between gross sequential motor skills and cognitive functions could provide added insight into atypical neurodevelopment in both disorders. Such studies would contribute to our understanding of the mechanisms underlying the relationship between motor and cognitive impairments. This would be of particular interest in the context of the ongoing debate regarding the links between motor and academic skills, such as reading acquisition [89–91].

Despite the shared motor impairments in all three groups compared to typically developing children, performance on the more complex bimanual out-of-phase coordination task differentiated AD from dyslexic and comorbid children, suggesting they had added difficulty with complex co-ordination. These findings are in line with studies that suggest motor deficits on more complex motor coordination in AD populations, which includes bimanual coordination [61]. They were slower than their controls and dyslexic children, and motor perseveration was more often seen compared to control, dyslexic and comorbid children. The latter type of error (tapping on the same plate twice) might be representative of difficulty inhibiting automatic responses which is a widely reported primary symptom associated with AD children [65, 68, 92]. This disinhibition is in line with Barkley’s hypothesis suggesting fragile inhibition control in AD populations. The added difficulty with asynchronized bimanual coordination is also in line with theories associating atypical frontal-based mechanisms with bimanual coordination [47, 48, 54, 55, 79, 93, 94]. Interestingly, a preliminary PET study on adults using the Leonard Tapping Task suggests that out-of-phase bimanual movements rely on the prefrontal cortex and the cingulate cortex, more so than for any other condition on the task [95]. However, our in-phase coordination task, which requires symmetrical bimanual coordination, did not differentiate between groups with neurodevelopmental disabilities. Our findings suggest that bimanual in-phase movements perhaps do not demand heavy reliance on frontal-based mechanism, concurring with reports that in-phase movements are less demanding because of our natural tendency to use both our limbs simultaneously [55]. Although impairments in inhibitory/frontal-based mechanisms may account for added difficulties in complex motor skills, an unexpected finding was that the COMB children were not also additionally impaired on the most complex coordination task, nor did they make more errors. Thus, deficits typically associated to an AD diagnosis, including behavioral inattention and hyperactivity, cannot be the sole factors in explaining the impairment observed in the complex out-of-phase coordination among our AD group. In addition, the AD children were the only ones with deficits on a manual dexterity task, which is in line with a study that suggested that fine motor skill deficits in AD are not associated to inattention, but rather to comorbid DCD [58]. We could hypothesize that the more severe motor problems in our AD group is associated with development coordination problems that were not identified by medical diagnosis. Interestingly, our AD group did comprise a higher number of children with co-morbid factors that are linked to atypical brain development [96]. These findings could indicate that while there is an overlap of motor deficits in both disorders, children with more widespread symptoms (reading and attentional) are not additionally impaired; rather the more severe and widespread impairments in our AD group could originate from the additive and interactive effects of the supplementary factors that hindered brain development. This is in line with studies with similar findings where the presence of comorbidity did not have an additional impact on task performances [12, 97]. In addition, many children with AD remain in regular schools and classes, as their symptoms are not severe enough to jeopardize successful learning. Our AD group might be representative of the more severe cases of AD, presumably because of other factors that hindered neurodevelopment and more specifically frontal-based mechanisms. Hence, our findings support a unifying framework, such as the Multiple Deficit Model [65], which posits that the overlap of neurocognitive symptoms in neurodevelopmental disorders can originate from interactive effects of genes and environmental risk factors, rather than from a single etiology. Our findings evidence the presence of a primary unifying impairment in gross sequential motor development in dyslexia and AD. Further studies that investigate the additive and interactive effects of multiple factors that hinder neurodevelopment as well as their association to more severe symptoms would be useful. We could better comprehend the overlap in these disorders and how to intervene optimally on functional impairments rather than on a specific diagnosis.

Although we predicted that participants with combined or separate disorders would generally show less improvement with repeated practice, we showed that they in fact improved similarly on most sequential conditions of the LTT over two short periods of 30 seconds. Hence, impairment in motor learning could not differentiate these children with typically developing peers. Motor sequence learning is characterized by rapid behavioral gains in the initial stages of motor practice, within a single session, whereas the later stages (slow, consolidation, automatization, retention) occur with practice over multiple sessions and offline mechanisms [98, 99]. Since our experiment was based on two conditions of 30 seconds within a single session, it is likely that the improvements observed here reflect changes in rapid motor adaptation rather than differences in skill consolidation or automatization. Further substantiating our findings is the evidence of the role of kinematics and dynamics in performance improvement in the SRTT paradigms [100]. Considering that our three experimental groups did not differ from their respective controls on measures of simple motor speed, it is possible that the improvements observed on the sequential conditions from Trial 1 to Trial 2 reflect adaptations in motor kinematics and dynamics rather than procedural learning per se. Nevertheless, our results show that AD and dyslexia do not generally impeach fast motor adaptations in the early stages of motor learning with the exception of the dyslexic participants that did not improve on the most complex coordination task. This could be the result of a compound effect of the complexity of the task and the well-documented impairments in procedural motor skill on motor adaptation. Further experiments that compare AD and dyslexia performances that readily differentiates motor adaptation and procedural learning over longer periods of time would be necessary to better understand how motor coordination learning is impacted by both neurodevelopmental disorders.

In conclusion, both disorders remain motorically challenged with gross sequential motor skills that rely on joint cognitive-motor control processes. Our study supports the hypothesis that motor skill impairment is a co-occuring symptom among both populations. The differences in motor skills with typically developing children is neither related to general motor slowness in responding or inability to adapt in the fast learning phase. Our findings support the Multiple Deficit hypothesis. The later theory unifies the considerable overlap of neurocognitive deficits between these developmental disorders and accentuates the importance of the additive and interactive effects of multiple factors to the understanding of the complex and dimensional symptomatology of developmental disorders. Our findings shed light on the importance of measuring motor skills at a young age for they can be useful indicators, among others, in identifying children who are at risk of neurodevelopmental disorders. Finally, early intervention with physical training to stimulate brain development is a field that offers an interesting approach, especially that a few recent studies have shown positive outcomes [101, 102], though it remains an area to be extensively explored. Further studies linking motor development and cognitive functions that target neurodevelopment as a complex multidimensional concept could help increase positive outcomes in terms of motor and cognitive interventions.

### Limitations

As mentioned in our *Clinical Measures* section, reading and spelling disabilities were defined as a performance of -1.3 SD on these two subtests. However, for the DYS group, the mean performance is 83.22 (11.69) on the reading subtest, which is equivalent to -1.1 SD (Table 1). This suggests that our DYS group performed slightly better than expected in terms of reading performances. We believe that this variability could be associated with children who have improved due to receiving special education, particularly because the WIAT-II subtest does not include a time limit. Our group could also include children with surface dyslexia rather than phonological dyslexia, which is assessed with irregular words rather than pseudo-word reading. As for the COMB group, results on the reading subtest is also higher than expected (Table 1). Again, this could be due to improvements in the performances associated with receiving special education or with the presence of children with subclinical reading and spelling difficulties, which warranted a comorbid medical diagnosis.

We obtained the confirmation of a medical diagnosis of AD, but no cognitive testing was done to confirm its presence and no differentiation was made between attention deficit disorder and attention deficit disorder with hyperactivity. As mentioned in our *Clinical Measures* section, the ADHD and ADD Index scores from the Conners-3 questionnaire were defined by a standard score over 60 (borderline range or higher). As shown in Table 1, the AD group’s mean performances are higher than the 60 T score cut off, though the large standard deviations indicate high variability in our group. We believe this could be due to factors such as medication, which was not controlled for, as well as improvement due to interventions offered in the specialized school. The same patterns of high variability on the Conners-3 scales were observed in our DYS and COMB group, though the mean group performances correspond to our cut-off criteria. The general variability in our groups suggests that they are comprised of children who are partially compensated or who have subclinical symptoms of either one of the disorders. However, this possible explanation is based on a limited number of tests, whereas the medical diagnosis is typically based on a complete cognitive evaluation which is required to attend the specialised school. Finally, participants with the dual diagnosis unexpectedly made fewer errors than their controls on certain conditions, which could suggest an artefact in our group. Gender was not controlled for when comparing between experimental groups.

In conclusion, our participant groups conceivably include children that are partially compensated in terms of academic or attentional symptoms. We believe, however, that the direction of the differences between group means on the reading and Conners-3 indexes, combined to the reported medical diagnosis, support our groups’ validity and represent the general population of children that attend specialised schools.

## Supporting information

S1 Dataset Minimal dataset—Marchand.xlsx. (XLSX)
